# A Scoping Review of Mental Health and Wellbeing Outcome Measures for Children and Young People: Implications for Children in Out-of-home Care

**DOI:** 10.1007/s40653-023-00566-6

**Published:** 2023-09-04

**Authors:** Paula Jacobs, Luke Power, Gavin Davidson, John Devaney, Claire McCartan, Pearse McCusker, Ruth Jenkins

**Affiliations:** 1 Faculty of Social Sciences, Social Work, University of Stirling, Colin Bell Building, Stirling, UK; 2 School of Social and Political Science, University of Edinburgh, Edinburgh, UK; 3 School of Social Sciences, Education and Social Work, Queen’s University Belfast, Belfast, UK; 4 Academic Support Librarian, University of Edinburgh, Edinburgh, UK

**Keywords:** Mental health, Measures, Care, Disability, Neurodivergence

## Abstract

**Purpose:**

One of the challenges for mental health research is the lack of an agreed set of outcome measures that are used routinely and consistently between disciplines and across studies in order to build a more robust evidence base for how to better understand young people’s mental health and effectively address diverse needs.

**Methods:**

This study involved a scoping review of reviews on consensus of the use of mental health and wellbeing measures with children and young people. We were particularly interested to identify if there are differences in measures that are recommended for children and young people with care experience including those with developmental disabilities.

**Findings:**

We identified 41 reviews, of which two had a focus on child welfare settings, three on childhood trauma and 14 focused on children and young people with developmental disabilities. Overall, our review highlights a lack of consensus and a diversity of measures within the field. We identified 60 recommended measures, of which only nine were recommended by more than one review.

**Conclusions:**

Our review highlights the need for greater agreement in the use of mental health outcome measures. While our review highlights that there is value in identifying measures that can be used with any child or young person, researchers need to take into account additional considerations when working with children and young people with care experience and those with developmental disabilities, to ensure measures are accessible and sensitive to their life experiences.

## Introduction

There is a growing recognition of a lack of agreement and consistency in the use of mental health measures in research and practice in relation to children and young people, which makes it difficult to compare research findings (Krause et al., 2021). For example, over 280 measures for depression have been developed since 1918 (Santor et al., 2006). How researchers assess mental health outcomes varies. Different measures reflect different mental health outcome domains that researchers decide to assess. Most commonly mental health is defined by looking at mental health problems, although there has also been an increase in positive mental health measures, utilising concepts such as wellbeing or quality of life (Losada-Puente et al., 2019). While it is important that there are robust measures for all aspects of mental wellbeing, common mental health problems and severe mental illness, the lack of consistency in the measures used means that important opportunities to compare findings across studies, settings and time are being missed. Additionally, the increase in the numbers of measures available has also led to concerns about the quality of measures that are used and the absence of independent evaluation of their psychometric properties (Addington et al., 2015; Howe et al., 2020), as well as if measures accurately reflect the different mental health outcome domains claimed (Krause et al., 2022).

The need for greater consensus has been highlighted by a number of initiatives. In 2005, the COSMIN initiative (COnsensus-based Standards for the selection of health Measurement INstruments) was set up by a multi-disciplinary team of international researchers to provide guidance on assessing and selecting suitable outcome measures. More recently the International Alliance of Mental Health Research Funders, the National Institute of Mental Health and the Wellcome Trust (Farber et al., 2020) have suggested a set of common data items and measures which should be routinely used in mental health research. These include: Age; Sex at Birth; WHO Disability Assessment Schedule (WHODAS) 2.0 (for adults); Patient Health Questionnaire (PHQ-9) (for adults); Generalised Anxiety Disorder Assessment (GAD-7) (for adults); and the Revised Children’s Anxiety and Depression Scale (RCADS-25) (for youth). The International Consortium for Health Outcomes Measurement (ICHOM) has recommended a standard set of outcomes specifically for child and youth anxiety, depression, obsessive compulsive disorder, and post-traumatic stress disorder (Krause et al., 2021) which are: the RCADS-25; the Obsessive Compulsive Inventory for Children (OCI-CV); the Children’s Revised Impact of Events Scale (CRIES); the Columbia Suicide Severity Rating Scale (C-SSRS); the KIDSCREEN-10; the Children’s Global Assessment Scale (CGAS); and the Child Anxiety Life Interference Scale (CALIS).

One of the reasons for a lack of agreement on which measures to use, is that different measures are often validated to be administered in specific groups and populations and within defined settings. Specific populations of interest include children and young people with care experience (also referred to as looked after children) including those with developmental disabilities. While there are increasing concerns about the mental health and well-being of children and young people in general (Frith, 2016), there are concerns in particular about looked after children (Bazalgette et al., 2015) and children with developmental disabilities, such as autism or ADHD (Sayal et al., 2018; Lecavalier et al., 2014). Children and young people in care are always, first and foremost, children and young people and there is a risk of othering those with care experience and/or developmental disabilities when viewing them as an entirely distinct and different group. Yet, some of their experiences will be unique and it is important for researchers to be aware of this. Young people who are looked after have consistently been found to have much higher rates of mental health difficulties than the general youth population, with almost half of looked after children (and three quarters of those living in residential group care) meeting the criteria for a psychiatric disorder in the UK (Fleming et al., 2021; McKenna et al., 2023). There are many reasons for this, including the adversities experienced by children before coming into state care, such as abuse, neglect, exploitation and poverty, along with the difficulties children may experience during their time in care, which can both add to and exacerbate their needs.

Given the accumulation of experiences, it is important to understand trajectories and outcomes of poor mental health and wellbeing, and recovery, for these young people to inform policy and practice. Reviews of the extant literature and research (Luke et al., 2014; NICE, 2015, 2021) have highlighted a number of challenges to ensure that the needs of looked after young people are better understood and addressed. The NICE (2015, 2021) Guidelines on Looked After Children and Young People concluded that further work was needed to develop robust methods for evaluating services. This included, for example, developing standardised, validated and reliable measures and robust tools to evaluate quality of life outcomes for use with all looked after children and young people from birth to 25 years, regardless of where they live.

Children and young people with developmental disabilities have also been found to be at greater risk of mental health difficulties due to an interplay of differences in individual functioning and environmental risk factors such as higher prevalence of bullying, experiences of stigma, lack of social inclusion and school exclusion (Sterzing et al., 2012; Honey et al., 2011; Emerson & Hatton, 2007). Additionally, there is now a growing recognition of the presence of developmental disabilities within the care population but this is often overlooked in mental health research with this group, despite potential implications for how such young people should be cared for, and supported (Banerjee et al., 2021). Recognising diversity in individual functioning and life experiences for children with care experience, including children with developmental disabilities raises the question of which measures are, can and should be used across populations, which need to be adapted and what population or domain-specific measures are needed.

### Aims and Objectives

The overall aim of the scoping review is to explore variability in the use of mental health outcome measures and to identify measures that have been recommended to be routinely used in research with children and young people in different contexts with a specific focus on children with care experience including those with developmental disabilities. The scoping review is part of a bigger project (blinded for peer review) and findings from this review informed the development of a Delphi study to identify and agree a common core set of measures to be used in mental health research with young people, who are care experienced. Additionally, as part of our project we conducted participatory work with young people and adults with care experience, including some with developmental disability, to help us think about how we should define and understand mental health, given the criticism that young people in out of home care are rarely asked about their perspectives on their own health (Smales et al., 2020). The scoping review was the first step of the process and aims to map the literature on the development and implementation of agreed outcome measures.

### Defining Mental Health Outcome Measures

For this review, outcome measures were defined as psychometrically validated measures of mental health. We aimed to take a broad conceptualisation of mental health and included related concepts such as wellbeing and quality of life, to capture clinical definitions, as well as broader social perspectives on mental health (Berghs et al., 2021). Additionally, it was important for the review team not to equate developmental disability with mental health problems and we decided not to include tools that facilitate a diagnosis of developmental disabilities such as autism or ADHD. The focus of this review is not on diagnosis, but on outcome measures that can be used in research to assess and understand young people’s mental health, identify risks or capture change over time.

## Methods: A Review of Reviews

Reviews of reviews are helpful in areas of research and practice that are rapidly growing and have an extensive evidence base that make the synthesis of primary studies too burdensome (Smith et al., 2011). An initial database search combining terms for measures, mental health and children in PsychInfo showed over 70,000 results of primary studies. The search results were then filtered to include systematic reviews published in the last 10 years. A further preliminary database search showed that there were several existing reviews that explored the use of mental health measures in research with children and young people with a focus on different age groups, settings and outcome domains. Thus, we made the decision to conduct a review of existing reviews to map recommendations for different populations and outcome domains.

### Research Questions

Our primary research question for the scoping review was: What outcome measures are currently used to assess the mental health and wellbeing of children and young people in research? Our aim was to map measures recommended by existing reviews for use in research with children and young people.

Sub-questions of interest were:

- How is mental health and wellbeing defined and what typologies and dimensions underlie existing measures?
- What outcome measures are used for children and young people in care and care-leavers? What outcome measures are used for children and young people with developmental disabilities?
- What are the age groups for which outcome measures have been designed and used?

### Search Strategy

The Joanna Briggs Institute (https://jbi.global/) recommends using PCC (Population – Concept – Context) to develop search strategies for scoping reviews, and the PCC format guided the development of our search strategy. The process was supported by an expert support librarian, who was a member of the research team (RJ). The search strategy was developed in PsycInfo, where the subject headings were likely to be the most detailed for mental health related terms, and the sensitivity of the search was tested using a set of papers already identified as relevant. The search strategy was then translated to Medline, Embase and ERIC. A combination of subject headings and keyword (free text) searches were used. The search was conducted between March and April 2021. An overview of our search terms can be found below (Table 1) and details of the full strategy with truncations and search filters can be obtained from the first author on request.

**Table 1:** Search terms

**Population**	**Concept**	**Context**
Children and young people	Mental Health and Wellbeing	Reviews of outcome measures
Child, Youth, Adolescents, Teenagers, Adult	Mental health, mental illness, mental disorder, emotional disturbances, psychological adjustment, psychological distress, social distress, isolation, loneliness, psychiatric disorder, well-being, global functioning, quality of life	Measure, assessment, psychometrics, rating, scale, screen, questionnaire, checklist, tool, self-report Review, meta-analysis, synthesis

### Eligibility Criteria

The following eligibility criteria were developed to guide the screening process:

- Is it a published review?
- Is it a review of measures?
- Is it a review of mental health measures?
- Does it focus on children and young people (0–26)?
- Is it available in English or German?
- Has it been published in the last 10 years (2011 to 2021)?

The age range was chosen to reflect current policy and practice recommendations, reflecting an understanding that the period of transition to adulthood can take several years after young people leave school. Additionally, mid-twenties have been identified as an age when most mental health conditions will have manifested (Kessler et al., 2007). Reviews that included studies with children/young people, as well as adult populations, were only included if they specifically referred to children or young people as a distinct group in their assessment and recommendation of measures.

We excluded specific populations such as children and young people with diabetes or terminal illness. Reviews were defined as following a systematic and transparent search process and included scoping, systematic and narrative reviews. The focus on English and German publications reflects the languages spoken by the research team, however, we recognise that the exclusion of other languages adds bias to the review.

### Screening Process and Data Extraction

**Fig. 1:**
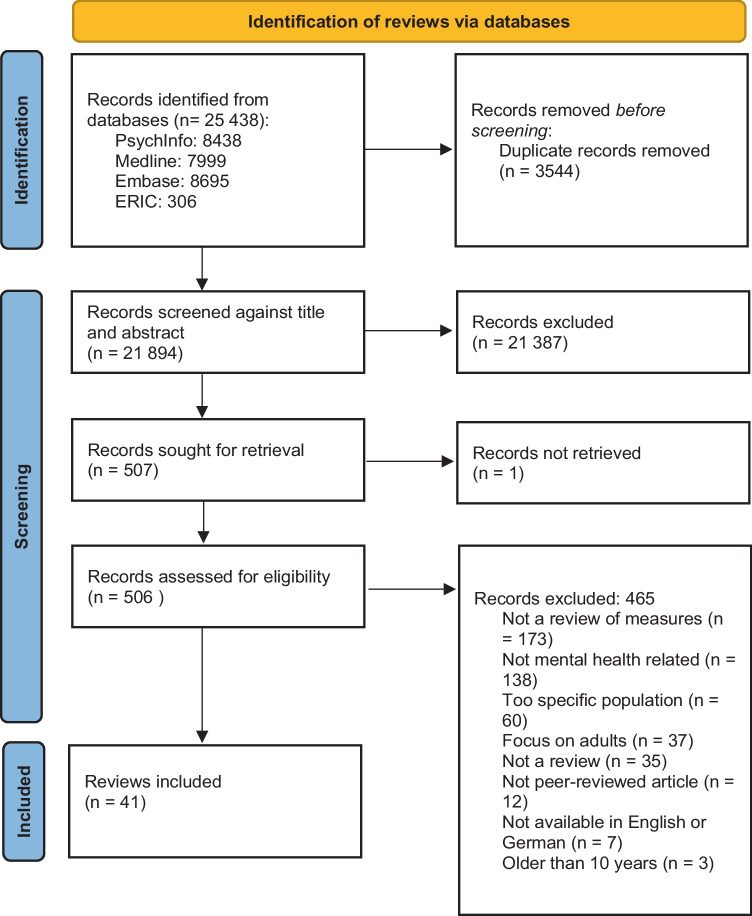
PRISMA flowchart

Overall 25,438 results were identified across the four databases and after removing 3,544 duplicates, 21,894 were screened against our eligibility criteria. 21,387 were excluded after screening all titles and abstracts and 506 were assessed for full-text eligibility, after we were unable to retrieve the full-text for one record. A team of six researchers conducted the screening (PJ, LP, CMC, JD, GD, PMC). 20% of results were assessed by two-reviewers at the title and abstract stage as a standardisation exercise, before moving to single reviewer screening. All full-text records were assessed independently by two reviewers. Conflicts were resolved by a third reviewer, and particularly difficult decisions were taken after discussions with the whole review team. An overview of the screening process can be found in the PRISMA diagram (Fig. 1).

The main reasons for exclusion at the full-text stage were reviews which did not meet our definition of being a systematic review of measures. This included reviews which focused on one or two specific measures and where the selection of those measures was not transparent or systematic. It also included reviews that reported the frequency of use of measures, but failed to provide an assessment of the psychometric properties or the acceptability or utility of identified measures. Additionally, 138 studies were identified as not primarily relating to mental health outcomes. This included reviews which focused only on physical health or physical functioning (e.g. mobility), IQ-tests or standardised diagnostic assessments.

41 reviews were deemed to meet our eligibility criteria. The data extraction process followed several steps. Firstly, extracting information about each included review study, including information about authors, country of origin, methods and the number of included studies and measures. Secondly, we identified recommended measures across the 41 reviews and information about each measure and the context of their use (recommended for which purpose, which setting, context and which age-group) was collated by two members of the research team (PJ and LP). To answer sub-questions of interest we used a framework of four mental health typologies to group reviews and measures (Slade, 2002). These were (i) condition-specific measures, (ii) behaviour associated with poor mental health such as self-harm or substance misuse, (iii) general mental health and (iv) positive mental health. We paid particular attention to reviews that discussed measures in relation to children with care experience and children with disabilities, comparing if different measures were recommended or if other differences were noticeable such as use of outcome domains.

## Results

The results section will provide an overview of included reviews, discuss findings in relation to outcome domains being used, identify recommended measures and lastly highlight findings in relation to children with care experience and children with developmental disabilities.

### Overview of Included Reviews

An overview of the 41 included reviews can be seen in the tables below and are presented in accordance to the four typologies (condition-specific measures, behaviour, general mental health, positive mental health). Tables include a description of the methods and aims of each included review, alongside a summary of key-findings and recommendations made by the authors (including use of measures with specific age ranges, populations or in specific settings). 21 reviews did recommend specific measures as part of their findings, while 20 reviews felt unable to provide a recommendation. Those reviews often noted that the choice of measure depends on specific research questions, aims, settings and groups of interest. Notably, most endorsed measures were recommended to be used in clinical or mental health specific settings, with none of the reviews exploring use of measures in community settings (such as schools). This seemed to be because authors felt that there was not enough evidence on the use of measures with diverse populations (Eklund et al., 2018). Additionally, few measures were identified that could be used in early childhood Tables 2, 3, 4, 5, and 6.

**Table 2:** Overview of reviews on condition/symptom-specific mental health measures

**Authors, Year and Country**	**Main focus**	**Method**	**Number of measures reviewed and number of studies included**	**Results and Recommendations**
Addington et al. (2015) **(USA)**	A review of screening instruments that identify people at increased risk of psychosis	Systematic review	17 measures reviewed 56 articles included	Does not provide a recommendation of specific measures Discusses strengths of a number of scales but highlights that the majority of measures is underexplored with poor validation
Bennett et al. (2017) **(UK)**	A review of measures that assess OCD in young people	Pragmatic review	17 measures reviewed 18 articles included	The authors identify the Y-BOCS as having the best psychometric properties but caution that additional factors should be considered when choosing a measure. For example, is this to diagnose, screen or to assess symptom severity/change?
Carnevale (2011) **(USA)**	A review of measures that can be used by school nurses to assess adolescent depression, with a link to economic evaluation	Integrative review	4 measures reviewed	The authors recommend Beck Depression Inventory-Youth (BDI-Y) and the Center for Epidemiological Studies-Depression Scale for Children The authors note that while all measures are reliable and valid, it is the affordability, ease of administration and the ability of the recommended tools that put them above the others
Groundhuis and Aman (2012) **(USA)**	A review of measures to assess anxiety in adolescents with autism	Comprehensive review	37 measures are reviewed 60 articles are included	The authors recommend the ADIS in conjunction with ACI-PL or CSI (for care giver report) or MACS or SCAS (for child report) as the best option for clinical and research application The authors note that the majority of measures do not do an adequate job in assessing anxiety in this population
Kreiser and White (2014) **(USA)**	A review of measures used to assess social anxiety in adolescents with autism	Comprehensive review	18 measures are reviewed 46 articles are included	Does not provide a recommendation of specific measures The authors note that while there are a number of measures used across a variety of studies, there is limited data on sensitivity and validity. This makes it difficult to make comparisons across studies
Lecavalier et al. (2014) **(USA)**	A review of measures that assess anxiety in youth with autism	Scoping review alongside a working group	12 measures are reviewed 38 articles are included	The authors recommend four measures that could be used in clinical trials: CASI-4R, PARS, MASC and Anxiety Diagnostic Interview Scale (ADIS)), and three measures that they deem potentially appropriate as an outcome measure: ADAMS, RCADS and SCARED The authors highlight the importance of considering the conditions under which these measures are being used
Tulbure et al. (2012) **(Romania)**	A review of measures used to assess social anxiety or phobia in youth	Systematic review	13 measures are reviewed 60 articles are include	The authors recommend 4 measures: The Social Phobia and Anxiety Scale for Children (SPAIC), the Social Anxiety Scale for Adolescents (SAS-A), Social Phobia Inventory—SPIN and the Liebowitz Social Anxiety Scale for Children and Adolescents—LSAS-CA
Wigham and McConachie (2014) **(UK)**	A review of measures to examine anxiety in children with autism in intervention studies	Systematic review	8 measures are reviewed 10 articles are included	The authors recommend 3 measures: The Children’s Anxiety Scale (revised version), the Revised Children’s Anxiety and Depression Scale and the Screen for Child Anxiety Related Emotional Disorders
Ashra et al. (2021) **(UK)**	A review of self-report measures of negative self-referential emotions for children and young people in non-clinical settings	Systematic review alongside a consultation of experts to identify measures	8 measures reviewed 12 articles included	The authors identify the Child Adolescent Perfectionism Scale (CAPS) and the Children’s Automatic Thoughts Scale (CATS) as the most rigorous in psychometric properties The authors highlight concerns about the appropriateness of measures and their sensitivity to change

**Table 3:** Overview of reviews on general Mental Health Measures

**Author, year and country**	**Main focus**	**Method**	**Number of measures reviewed and number of studies included**	**Results and recommendations**
Bradford and Rickwood (2012) **(Australia)**	A review of psychosocial measures for use with the general population of young people	Systematic review	31 measures are reviewed 89 articles are included	No recommendation is provided The authors highlight the acceptability of self-reported measures to young people. Moreover, they state that most psychosocial measures can improve rates of disclosure and enhance engagement
Crowe et al. (2011)	A review of measures of social functioning in children and young people	Systematic review	86 measures reviewed No information about the number of articles included	The authors discuss strengths and weakness of identified measures. No specific recommendation is provided
Deighton et al. (2014) **(UK)**	A review of child self-reported measures for mental health and well-being	Systematic review and stakeholder consultation	11 measures are reviewed No information on the number of studies included	The authors discuss the strengths and limitations of all 11 measures but do not provide a specific recommendation
Halle and Darling-Churchill (2016) **(USA)**	A review of measures of youth social and emotional development	Critical review	75 measures are reviewed No information on the number of studies included	The authors recommend the Devereux Early Childhood Assessment Clinical Form (DECA-C), the Social Skills Rating System (SSRS) and the Infant Toddler Social Emotional Assessment (ITSEA) if assessing the social and emotional development in the earliest years of life
Kwan and Rickwood (2015)	A review of mental health measures for youth aged 12-25yrs	Systematic review	29 measures are reviewed 185 articles are included	The authors identify 8 measures that have been used across the age-range. They discuss the strengths and limitations of all 8 measures but do not provide specific recommendations
McCrae and Brown (2017) **(USA)**	A review of measures that can be used for welfare involved youth	Systematic review	24 measures are reviewed 24 articles are included	The authors recommend 8 measures: ASQ-SE, BITSEA, PKBS-2, PPSC (Toddlerhood to preschool) and PSC, SDQ, ECBI, ISSC (school age to adolescence)
Janssens et al. (2016) **UK**	Reviews PROMS to used with children and young people with neurodisabilities	Systematic review The review is complemented by qualitative research with neurodiverse young people, their parents and a Delphi survey of health professionals	12 measures reviewed 48 studies included	Does not provide a recommendation of specific measures Strengths and weaknesses of included PROMs are highlighted The review highlights a lack of comprehensive evaluation of identified PROMs, with responsiveness and measurement error being the least studied. The authors note a lack of evidence on responsiveness and measurement error, limiting the use of PROMs to measure change over time
Eklund et al. (2018) **USA**	Review of trauma screening tools to be used with children and young people in school settings	Systematic review	18 measures reviewed No information about the number of included studies	Does not provide a recommendation of specific measures The authors note little psychometric evidence to support the use of measures in schools and emphasise the lack of a broader screening school that is able to capture diversity in school populations
Atazadeh et al. (2019) **(Iran)**	A review of measures of post trauma in children	Systematic review	27 measures are reviewed in detail No information about the number of included studies	Does not provide a recommendation of specific measures Provides information about psychometric properties and the use of different measures
Zima et al. (2019)	A review of child measures for use in a state-wide outpatient mental health program	Review and modified Delphi study	15 measures are reviewed	The authors recommend the Paediatric Symptom Checklist (PSC) Additionally, authors note that the Delphi panel recommended the Achenbach System of Empirically Based Assessments and the Strengths and Difficulties Questionnaire for a number of domains

**Table 4:** Overview of reviews on Positive Mental Health Measures

**Authors, Year and Country**	**Main focus**	**Method**	**Number of measures reviewed and number of studies included**	**Results and Recommendations**
Satapathy et al. (2020) **(India)**	A review of resilience tools that can be used with children and adolescents, that have experienced traumatic life events	Systematic review	12 measures reviewed 12 studies included	Does not provide a recommendation of specific measures Notes that none of the measures was originally developed for children and adolescent with histories of trauma, but that the Child and Youth Resilience Measure and Connor-Davidson Resilience Scale included small samples of children from welfare homes Includes a more detailed discussion of cultural adaptation with focus on the Asian, particularly Indian, context
Bentley et al. (2019) **(UK)**	A review of self-reported measures for adolescents’ general health/ wellbeing	Systematic review	16 measures are reviewed 27 articles are included	The authors recommend the YOQL-R or Y-OQ (30.1) The authors caution that there needs to be further robust testing of the psychometric properties of adolescent measures
Rose et al. (2017) **(USA)**	A review of measures of adolescents’ mental wellbeing	Systematic review	11 measures are reviewed 689 articles are included	The authors do not provide a specific recommendation The authors note that only 4 of the measures were developed for adolescents and highlight the importance of measurements being culturally and conceptually relevant within groups of adolescents
Tsang et al. (2012) **(Hong Kong)**	A review of measures that assess the psychosocial wellbeing of adolescents	Systematic review	17 measures are reviewed 29 articles are included	The authors do not make a specific recommendation The authors note that strengths-based measures could potential link up assessment and promotion of wellbeing in adolescents’ homes, schools and communities
Rosanbalm et al. (2016) **(USA)**	A review of wellbeing measures used with children in welfare settings	Narrative review of four specified tools, identified as the most used in child welfare settings. The review is complemented with interviews of the tools’ developers and practitioners	4 measures reviewed No information about the number of included studies is provided	Does not provide a recommendation of specific measures The authors conclude that overlap in content and utility across the four tools is high and identify strengths and weaknesses for each tool
Davis et al. (2018) **(Australia)**	Reviews Quality of Life measures that can be used by service providers to assess the holistic wellbeing of children with disabilities and incorporate a rights-based perspective	Systematic review of reviews	20 measures reviewed 2 systematic reviews included	KIDSCREEN and KINDL are deemed to incorporate a rights-based approach, to be suitable for use in a service provision settings, as well as being sensitive to change The authors note that many generic QoL instruments focus on functioning, rather than wellbeing and caution that in the context of disabilities, functional limitations do not necessarily correlate with lower QoL
Ikeda et al. (2014) **(New Zealand)**	Reviews measures that have been used in research to measure QoL or HRQoL in autistic children and young people	Systematic review	8 measures reviewd 13 studies included	The PedsQL is recommended as a QoL measure to be used with autistic children and young people The authors note the importance of self-report measures to listen to children’s voices. Additionally, the authors caution that autistic children and young people might struggle completing self-report instruments, which were not developed with autistic children in mind
Losada-Puente et al. (2019) **(Spain)**	Reviews QoL measures with a special focus on their use with adolescents with disabilities to improve educational practices	Systematic review	9 measures reviewd 38 studies included	Does not provide a recommendation of specific measures. The authors note that the choice of measure will depend on the purpose of each individual study The authors recommend combining subjective and objective measures
Mierau et al. (2020) **(Netherlands)**	Reviews generic QoL measures that can be used with children and adolescents with mental health problems and have a link to economic evaluation	Systematic review of reviews	22 measures reviewed 29 reviews included to identify measures, alongside a review of 30 studies on the application of these measures in research with children with mental health problems	Measures are recommended for different age-groups and in relation to self and proxy-reporting. This includes the CHIP, CHQ, PEDSQL and KIDSCREEN
McConachie et al. (2015) **UK**	Reviews measures that assess progress and outcomes in autistic children up to the age of 6	Systematic review Findings were sense-checked with autistic young people, parents, clinicians and researchers	12 measures are reviewed in detail 180 studies included To identify measures, alongside a review of 122 studies to assess the psychometric properties of included measures	Does not provide a recommendation of specific measures The authors note a lack of evidence of the ability of measures to capture change over time The authors stress the importance to involve children and families in planning and developing measurement tools

**Table 5:** Overview of reviews on Behaviour Measures

**Authors**	**Main focus**	**Method**	**Number of measures reviewed and number of studies included**	**Results and Recommendations**
Carter et al. (2019)	A review of measures that identify immediate risk of self-harm and suicide in children and young people in clinical settings	Scoping review	22 measures reviewed 22 studies included to identify measures, alongside a review of 62 studies on the application and psychometric properties of these measures	The authors identify the Columbia Suicide Severity Rating Scale (C-SSRS) as the most rigorously studied and with a good performance across psychometric domains However, the authors highlight a lack of applicability in a variety of countries and settings and a lack of developmental considerations of different age groups
Harris et al. (2019**)** **UK**	Reviews measures that predict future self-harm and suicide in adolescents in clinical settings	Systematic review	10 measures reviewed 11 studies included	Does not provide a recommendation of specific measures Authors note that predictive validity statistics varied greatly across measures, with some showing high sensitivity but low predictor value, or the other way around
Howe et al. (2020) **Canada**	Reviews measures that assess suicide risk in children and young people with autism in research and clinical practice	Systematic review	10 measures reviewed 17 studies included	Does not provide a recommendation of specific measures The authors note a lack of independent validation studies and a lack of testing measures with autistic young people. They stress the need to distinguish between suicide risk and self-injurious behaviours which can serve a self-stimulatory function for some autistic people
Matson and Cervantes (2014)	Reviews measures that assess aggression in autistic people (including adults and children)* * Although the focus is not exclusively on children and young people, the majority of identified measures is applied in populations of 21 or younger	Systematic review	42 studies included	Does not provide a recommendation of specific measures The authors note an increase in the development of measures and specialization with autistic people in recent years. Different measures will suit different research questions and populations (e.g. children, with or without intellectual disability, different settings)
Hanratty et al. (2015) **(USA)**	A review of measures of behaviour problems in young children with autism	Systematic review	6 measures reviewed 15 studies included	The Child Behavior Checklist (CBCL) and the Home Situations Questionnaire—Pervasive Developmental Disorders version (HSQ-PDD) are recommended but a number of weaknesses are identified The authors note difficulties of measures to be able to differentiate between autism-related behaviour from other problems measures, including anxiety
Hall et al. (2016)	To review continuous performance tests and objective measures that diagnose and monitor medication management for children with ADHD in clinical contexts	Systematic review	5 CPT measures reviewed 2 objective measures reviewed 60 studies included for CPT measures 25 studies included for objective measures	Does not provide a recommendation of specific measures but the authors note that that the TOVA and GDS had the largest evidence base for clinical utility The authors recommend the combination of objective measures of activity and CPTs
Erford et al. (2018) **USA**	Reviews outcome measures to be used with disruptive children and young people within a counselling context	Review and meta-analysis	6 measures reviewed 50 studies included	The authors recommend the new ASEBA subscales and highlight its multiple informant versions, but recommend the Conners-3 long versions for assessment of ADHD, oppositional defiant disorder and conduct disorders The authors state that low power requires caution in interpretation of their findings
Pilowsky and Wu (2013) **USA**	Reviews substance use screening measures to be used with adolescents in primary care settings	Critical review	7 measures reviewed 35 studies included	The CRAFFT is recommended as the most thoroughly studied and most used instruments with adolescents
Hock et al. (2015)	Reviews measures of family engagement and assesses their utility for adolescent substance use treatment settings	Systematic review	8 measures reviewed	Does not provide a recommendation of specific measures The authors note that few measures cover different engagement domains (attitudinal, affective, behavioural). Only the CASII/CALOCUS and VTAS-R, are identified as having items that cover all three domains

**Table 6:** Overview of reviews on various Measures

**Authors**	**Main focus**			**Method**		**Number of measures reviewed and number of studies included**		**Results and Recommendations**			
Krause et al. (2021)	A review of various outcome measures to be used by those providing care for children and young people			Scoping review alongside a working group and a Delphi process		31 measures reviewed 257 articles included		The following measures are recommended: RCADS-25 for Anxiety and Depression, CRIES-8 (children and youth) and CRIES-13 (parent report) for PTSD, OCI-CV for OCD, C-SSRS for suicidal thoughts and behaviour, KIDSCREEN-10 as a generic measure of global functioning, CGAS as a brief clinician-rated measure of global functioning, CALIS for anxiety-related impairments of functioning The authors recommend that the set should be used alongside the anxiety and depression standard set for adults with clinicians selecting age-appropriate measures			
Becker-Haimes et al. (2020) (USA)	A review of various measures used to assess the mental health of youth			Systematic review		95 measures are reviewed: 12 for overall mental health, 11 for anxiety, 13 for depression, 12 for disruptive behaviour, 7 for trauma, 12 for eating disorder, 6 for suicide, 6 for bipolar, 3 for psychosis and 13 for substance used No information about number of articles		The review recommends 21 measures across different areas. These include Ohio Scales, SDQ, PSC, YP-CORE (general mental health); VADTRS, SNAP-IV, SWAN, IOWA Conners (disruptive behaviours); BITE, EDDS (disordered eating); ABUSI (suicide and self-harm); CPTCI (traumatic stress); CMRS-P (mania); RCADS/RCADS-P, PANAS-C, PHQ-9, MFQ (depression); RCADS/RCADS-P, SCARED, SCAS (anxiety) The authors state that few “excellent” measures were found for eating disorder, suicidality, psychosis and substance use			
Newton et al. (2017) Canada	Reviews measures to screen and diagnose mental health and substance problems be used with children or adolescents in emergency departments			Systematic review		18 measures reviewed 14 studies included		The ASQ is recommended in relation to screening for suicide risk due to its high sensitivity, the HEADS-ED in relation to general screening, and the DSM-IV instrument in relation to substance use (two-items) due to its good sensitivity and specificity, as well as highlighting that all three are easy to use			

### Dimensions of mental health

The included reviews were based on different concepts of mental health. These included (i) nine reviews of symptom and condition-specific measures (e.g. depression, anxiety, psychosis), which focused on the presence of symptoms, were often closely related to diagnostic criteria and used in clinical settings; (ii) nine reviews that focused on behaviour associated with poor mental health, including substance use, aggression, disruptive behaviour, self-harm and suicide; (iii) ten reviews that focused on general mental health measures, combining an assessment of multiple dimensions such as cognition, social and emotional development and functioning in different environments; and (iv) ten reviews that utilised positive mental health perspectives, assessing wellbeing, quality of life and resilience through concepts such as life satisfaction, participation, sense of belonging in combination with consideration of the impact of environmental factors such as relationships, or housing. Both general mental health measures and positive mental health measures included examples of one-dimensional measures, providing an overall score across domains, as well as multi-dimensional ones considering individual domains alongside each other. Three of the reviews had a wider scope and reviewed measures across typologies (Becker-Haimes et al., 2020; Krause et al., 2021; Newton et al., 2017). Examples of measures for each typology included the Revised Children's Anxiety & Depression Scale as a condition-specific measure for depression and anxiety; the Columbia Suicide Severity Rating Scale which evaluates severity of behaviour and ideation; the Paedtriatic Symptom Checklist, which involves an assessment of psychosocial problems, as well as overall functioning including school and peer relationships; and KIDSCREEN as an example of a measure of wellbeing that includes questions about physical and psychological wellbeing, mood and emotions, autonomy, home life, relationships, social support and school.

Interestingly, we had initially thought that measures of wellbeing and quality of life would reflect a more positive perspective to mental health. However, during the review and data extraction process we became aware that authors were highlighting that some wellbeing and quality of life measures are often applied in studies that take a deficit-view to highlight limitations or difficulties (Davis et al., 2018; Mierau et al., 2020). Thus, wellbeing and quality of life measures were often found to be used within narratives that focus on psychopathology, rather than identifying what helps children and young people to be well (Losada-Puente et al., 2019).

### Overview of recommended measures

Overall, 60 measures were recommended by 21 reviews. Interestingly, a number of reviews had the same areas of interest (e.g. measures of anxiety or risk of suicide) but came to different conclusions and recommendations. This appeared to be because of different foci in relation to the exact purpose of the measures or their use with different age-groups or populations and different priorities in the assessment of the measures. For example, some reviews had a stronger consideration of predictive values when making recommendations in relation to the identification of early risk (Harris et al., 2019), while others focused on the sensitivity of measures in relation to using them as screening tools or to capture change over time (Newton et al., 2017). Reviews assessing the use of measures in schools or clinical practice tended to include a stronger consideration of their utility and acceptability to children and young people (McConachie et al., 2015; Rosanbalm et al., 2016). Yet, it was still striking how little consistency there was across reviews on which measures to use. For example, we identified 15 different recommended measures in relation to the assessment of anxiety. Similarly, Bear et al. (2020) identified 15 different measures in their systematic review of outcome measures of anxiety and depression in young people.

To narrow down the list of recommended measures we looked at which measures were recommended by more than one review. Only nine measures were recommended more than once and these are presented in the table below (Table 7). An overview of the full 60 measures including information on recommended populations, settings and number of items, can be found in Appendix 1. The Revised Children's Anxiety & Depression Scale (RCADS, long and short version) was the most recommended measure with four reviews recommending it as a measure for anxiety and depression and it is also included in the set of measures recommended by ICHOM and the Wellcome Trust. Reviews recommended it for the age range of 6 to 18 years, within clinical and community settings. Strengths that were noted included its use in different cultural contexts, but Krause et al. (2021) noted that they did not find evidence of its sensitivity to change.

**Table 7:** Measures

Focus		Recommended population(s)	Purpose and domains	Informant	Number of items/ length (minutes)	Free	Recommended by
GENERAL MENTAL HEALTH	Paediatric Symptom Checklist (PSC; Jellinek et al., 1988)	4–16 years (PSC), Children and young people in mental health services, Care experienced children and young people	Assessment measure, Psychosocial problems and overall functioning (including school and peer relationships)	Proxy (caregiver)	35 items 9–12 min	Free	Zima et al. (2019), McCrae and Brown (2017) and Becker-Haimes et al. (2020)
	Strengths and Difficulties Questionnaire (SDQ; Goodman, 1997)	3–16 years, Community mental health settings, Care experienced children and young people	Assessment measure, Prosocial behaviour and psychopathology	Self and proxy-report	25 items 5 min	Free	Becker-Haimes et al. (2020) and McCrae and Brown (2017)
QUALITY OF LIFE, HEALTH RELATED QUALITY OF LIFE AND WELLBEING	KIDSCREEN (EU-consort 2001–2004)	8–18 years, Children and young people in clinical care, Youth with disabilities, Validated in children with ADHD	Assessment and outcome measure, Covers wellbeing, emotions, cognition, social relationships (home and school), autonomy and activities. For initial assessments, as well as interventions (change)	Self and proxy-report	52, 27 or 10 5 to 20 min	Free	Krause et al. (2021), Davis et al. (2018) and Mierau et al. (2020)
	Pediatric Quality of Life Inventory (PedsQL; Varni et al., 1999)	8–18 years, Young people in mental health services, Poor quality in use with younger children (Mierau et al., 2020), Validated with autistic youth and children with ADHD and intellectual disabilities (Mierau et al., 2020)	Assessment measure, Covers physical, emotional, social and school functioning	Self and proxy-report	23 4 min		Ikeda et al. (2014) and Mierau et al. (2020)
ANXIETY AND DEPRESSION	Revised Children's Anxiety & Depression Scale (RCADS; Ebesutani et al., 2010, 2012)	6–18 years, Children and young people in mental health services (clinical and community settings), Children and young people with autism	Outcome and assessment measure, Anxiety and Depression	Self and proxy-report	25 or 47 5 to 10 min	Free	Krause et al. (2021), Lecavalier et al. (2014), Becker-Haimes et al. (2020) and Wigham and McConachie (2014)
	Anxiety Disorders Interview Schedule (ADIS, Silverman & Albano, 1996)	6–18 years, Children and young people with autism	Recommended as a measure to detect treatment effect (no longer meeting diagnostic criteria) and for characterization of research participants. Administration burden make it unsuitable as a repeat measure	Clinician interview child or/and caregiver	Diagnostic semi-structures interview for anxiety disorder (DSM-IV)	Not free	Lecavalier et al. (2014); Groundhuis and Aman (2012) recommend it in combination with other measures
	The Screen for Child Anxiety Related Emotional Disorders (SCARED; Birmaher et al., 1997)	6–17 years, Community mental health settings, Children and young people with autism	Assessment and outcome measure of anxiety Assessment and outcome measure of anxiety	Self and proxy	41 items	Free	Lecavalier et al. (2014), Wigham and McConachie (2014) and Becker-Haimes et al. (2020)
	Spence Children’s Anxiety Scale (SCAS; Spence, 1997)	8–15 years, Community mental health settings, Children and young people with autism	Assessment and outcome measure of anxiety	Self and proxy	44 items	Free	Becker-Haimes et al. (2020), Wigham and McConachie (2014
SUICIDE/ SELF-HARM	Columbia Suicide Severity Rating Scale (C-SSRS; Posner et al., 2011)	12–18 years, Children and young people in mental health services	Assessment measure, Evaluates the severity of behaviour and ideation	Self-report	Includes 6 questions	Free	Krause et al. (2021) and Carter et al. (2019)

Two measures were recommended by three reviews. The Paediatric Symptom Checklist (PSC) was recommended as a general mental health measure, assessing internalising, externalising and general mental distress for the ages 4 to 16 years (Becker-Haimes et al., 2020; Zima et al., 2019; McCrae & Brown, 2017). The Screen for Child Anxiety Related Emotional Disorders (SCARED) was recommended as another assessment and outcome measure of anxiety, with reviews highlighting its strong psychometric properties (Becker-Haimes et al., 2020; Lecavalier et al., 2014;).

All other measures were recommended by two reviews. This included a further two anxiety measures. The Anxiety Disorders Interview Schedule (ADIS), was recommended specifically to be used with autistic children for the ages of 6 to 18 years, to detect treatment effect (no longer meeting diagnostic criteria) and for characterization of research participants. However, Lecavalier et al. (2014) noted that administration burden makes it unsuitable as a repeat measure.

The Spence Children’s Anxiety Scale (SCAS) was recommended to be used in community mental health settings, as well as with autistic children and young people for the ages 8 to 15 years (Becker-Haimes et al., 2020).

KIDSCREEN was recommended as a wellbeing and quality of life measure for young people between 8 and 18 years. Identified strengths included its sensitivity to change over time, as well as accessibility and ease of use in practice, having been developed with input from children, young people and their families (Davis et al., 2018; Krause et al., 2021). The Pediatric Quality of Life Inventory (PedsQL) was recommended as another quality of life measure for the ages 8–18 years. Limitations included its poor quality when used with younger children (Mierau et al., 2020), as well as its high cost (Davis et al., 2018).

The Strengths and Difficulties Questionnaire (SDQ) was recommended as a general mental health measure for the ages 3–16 years, with Becker-Haimes et al. (2020) emphasising evidence for its use as a routine measure of progress over time.

Lastly, the Columbia Suicide Severity Rating Scale (C-SSRS) was recommended to evaluate the severity of suicidal behaviour and ideation. Krause et al. (2021) noted that there had been no validation of the C-SSRS recent self-report measure to be used with children and young people, but that the clinician-rated C-SSRS had strong evidence of good internal consistency, inter-rater reliability, and sensitivity to change in adolescent samples.

### Recommendations in Relation to Children and Young People with Care Experience and Those with Developmental Disabilities

We identified two reviews with a focus on children and young people with care experience in relation to general mental health measures and measures of wellbeing (McCrae & Brown, 2017; Rosanbalm et al., 2016) and three that focused on trauma related experiences in relation to general mental health (Atazadeh et al., 2019; Eklund et al., 2018) and resilience (Satapathy et al., 2020).

In relation to developmental disabilities, we included five reviews, which focused on symptom or condition-specific measures, which all related to autism and anxiety (Kreiser & White, 2014; Lecavalier et al., 2014; Wigham & McConachie, 2014; Tulbure et al., 2012; Grondhuis & Aman, 2012), three reviews focused on the assessment of specific behaviours, which included aggression and self-harm in autism (Hanratty et al., 2015; Howe et al., 2020; Matson & Cervantes, 2014), one discussed medication management and symptom changes in ADHD (Hall et al., 2016), three focused on quality of life and general mental health outcomes in relation to disabilities as a general concept (Davis et al., 2018; Janssens et al., 2016; Losada-Puente et al., 2019), and two focused on autism and quality of life (Ikeda et al., 2014; McConachie et al., 2015). This shows that while developmental disabilities include a very diverse group of children and young people, there appears to have been greater focus on autism over other disabilities.

Only one of the reviews that focused on children and young people with care experience made recommendations, which included the SDQ and the PSC, which were also recommended to be used with young people in mental health settings. Satapathy et al. (2020), in their review on resilience measures, further discussed that the Child and Youth Resilience Measure and Connor-Davidson Resilience Scale included small samples of children from welfare homes. Three anxiety measures (RCADS, SCARED, SCAS) were recommended for young people in the general population as well as autistic youth, with the ADIS being specifically recommended for autistic children and young people (Lecavalier et al., 2014; Groundhuis & Aman, 2012). Additionally, Lecavalier et al. (2014) highlighted that one study had evaluated the use of RCADS with autistic children (Hallett et al., 2013), which has since been repeated adding further support for its use with autistic children and young people (Sterling et al., 2015). The KIDSCREEN (long and short versions) was recommended for use with children and young people in clinical care, youth with disabilities and children with ADHD. The PedsQL was recommended to be used with young people in mental health services, as well as autistic youth, young people with ADHD and intellectual disabilities (Mierau et al., 2020).

All reviews on children and young people with care experience focused on general mental health or positive mental health measures. This reflected a view that holisitic assessments would help capture the complexity of experiences in this population. Additionally, in relation to oucome domains, all reviews on children and young people with care experience and some of the reviews that focused on children and young people with developmental disabilities highlighted the value of measures that included an assessment and questions on strengths alongside difficulties or deficits, as well as assessments that included a consideration of environmental factors alongside individual ones (Davis et al., 2018; McConachie et al., 2015; McCrae & Brown, 2017). Authors argued that, for both populations, environmental factors often contribute to and sustain poor mental health, and that it is important for researchers and practitioners to understand and capture poor mental health as a response to trauma and experiences of social exclusion or stigma (Davis et al., 2018; Ikeda et al., 2014; McConachie et al., 2015; McCrae & Brown, 2017). Similarly, two of the reviews which involved children and young people in the assessment process of measures with a focus on autism (McConachie et al., 2015) and developmental disabilities (Janssens et al., 2016) highlighted discrepancies between what was being measured and what children, young people or their families identified as important to them, as well as highlighting the importance of measures being accessible. This included a dominant focus on deficits and difficulties, overlooking the strengths and abilities of children and young people.

## Discussion

Having identified over 60 recommended measures, only nine were recommended by more than one review which adds to the evidence for the lack of consensus on the use of mental health measures in research with children and young people. Across the included reviews the tension between having specific measures that are validated for use in particular settings, with specific age groups and populations, that can also address defined research aims and questions (such as measuring change over time, having predictive power) was evident. Reviews which focused on developmental disabilities emphasised that many measures were not designed with children and young people with disabilities in mind, which was also true in relation to children and young people with care experience and those who have experienced adversities (McCrae & Brown, 2017; Satapathy et al., 2020). Yet, authors argued that instead of developing new measures it can be more helpful to adapt and develop existing measures to build on existing knowledge. This allows researchers to make comparisons, while remaining aware of specific needs and circumstances, particularly as mental health tools can subsequently be validated for their use with children or young people with developmental disabilities (Biederman et al., 2005; Sterling et al., 2015) or those with care experience. Similarly, Krause et al. (2021) argue for the piloting of existing measures in new populations and contexts to adapt or exchange them in light of new evidence and knowledge.

Only two of the nine measures that were recommended by more than one review were recommended to be used with young children under 6 years of age (proxy versions). These measures (the PSC and SDQ) were also recommended to be used with children and young people in care. None of the nine measures that were recommended more than once were recommended for both children and young people with care experience and children and young people with developmental disabilities, neglecting the intersectionality of both (Gajwani & Minnis, 2023). A focus on autism over other developmental disabilities was noticeable and when considering intersections of care experience and developmental disabilities it will be important for future research to consider other conditions such as FASD and ADHD (Gajwani & Minnis, 2023).

Next to a lack of consensus of which measures to use, our review also identified a lack of consensus of how to assess or review existing measures and how to report psychometric properties. Reviews differed in their reporting of psychometric properties. For example, there were differences between reviews only reporting psychometric data from the original studies of the development of measures, while others synthesised information from subsequent independent studies. This made it difficult to include and compare information on psychometric data. There are existing guidelines on the reporting of psychometric properties, including frameworks by the American Psychological Association (Gehrig, 2019), and our findings point to a poor use of those frameworks. The importance of having consensus in how we assess outcome measures is furthermore highlighted by the COSMIN initiative, which provides guidelines and standards, and to which a number of the reviews in this study referred. Alongside reliability, validity and responsiveness, COSMIN advocates for a consideration of interpretability, which is also sometimes referred to as acceptability or utility. There was less consideration of utility and acceptability within our review of measures, compared to reliability, validity and responsiveness. Similarly, in their review of psychosocial interventions for maltreated children and young people Macdonald et al. (2016) found that researchers often fail to consider issues of accessibility and acceptability. While high quality and evidence-based research relies on reliable and valid outcome measures, researchers have started to pay attention to their acceptability as well. This reflects the importance that children and young people understand the questions and items asked and that they feel those reflect their experiences. Thus, alongside psychometric assessments researchers have started to involve service users and experts by experience to evaluate and adapt assessment and treatment processes (Krause et al., 2021; Macdonald et al., 2016). Equally, the reviews focusing on developmental disabilities also highlighted the importance of involving children and young people (Davis et al., 2018). Reviews with a focus on autism discussed the significance of adapting self-report items and questions to ensure measures are accessible and inclusive (Ikeda et al., 2014; McConachie et al., 2015). This will be similar in relation to children and young people with care experience. Questions around family life and relationships need to be able to capture the diverse experiences of children and young people who might have experienced multiple placement changes, family conflict and for whom the concept of ‘family’ might be ambiguous or sensitive. Research with children and young people in care has highlighted that other significant people such as teachers, sports coaches or friends can be their closest relationships (Frederick et al., 2023), and it might be important to be more inclusive in the assessment process, asking young people who they trust, and to identify who the key people in their life are. As McCrae and Brown (2017) suggest: *“Perhaps more of an issue than choosing screening tools with valid scientific properties is ensuring that instruments meet the needs of children and families.” (p. 784).* Involving children and young people with care experience in the process of adapting and assessing measures is an important next step (Smales et al., 2020). This will also help us to understand children and young people’s experiences of assessment processes and in how far they are able to help researchers and practitioners to understand their experiences and facilitate engagement (Bradford & Rickwood, 2012; Tsang et al., 2012).

Additionally, in relation to children and young people with care experience and their families it is important to understand that the process of conducting assessments is a relational one. Children might find it difficult to engage in overly restrictive processes and may mistrust professionals due to past experiences (MacCrae & Brown, 2017; Macdonald et al., 2016). Similarly, McConachie et al.’s (2015) work with autistic young people and professionals stressed how the use of measures that take a deficit view can impact negatively on the relationship and engagement between professionals who undertake a problem focused assessment with children and young people. Previous research has shown that clinical definitions of mental health can often be restrictive and not fully supported by the experiences of young people themselves or research (Macdonald et al., 2016; Zhang & Selwyn, 2019). This highlights the importance to not only think about **which** measures are used, but also if **what** is being measured matters to children and young people, **how** measures are used and **how** the assessment process impacts on children and young people.

## Conclusion

It is hoped that this review adds to the ongoing consideration and development of approaches to more effectively and consistently measure the mental health outcomes of young people, including those that are care experienced and those that have developmental disabilities. Research designs which enable links across settings and countries will facilitate comparison, although there should be some caution about what is appropriate to compare. It should also be acknowledged that these are not all of the outcomes that may be important, but by seeking to use an internationally agreed set of mental health and well-being measures in research involving young people there is a greater likelihood of building a comprehensive understanding of the diversity and totality of needs, and how to meet these needs effectively. While a tension remains between having recommended outcome measures to enable consistency in the application of questions, items and scores, and ensuring that measures are sensitive to the contexts of different populations and settings, we agree with Krause et al. (2021) that it will be important to create greater consensus and to understand mental health measures as evolving tools that are co-owned and co-produced with those that should benefit from them, while upholding the value of reliability, validity and responsiveness.

## Data Availability

Details of the search and review process can be obtained by contacting the corresponding author.

## Appendix

**Table 8:** Measures

**Focus**		**Recommended population(s)**	**Purpose**	**Informant**	**Number of items**	**Free**	**Recommended by**
GENERAL MENTAL HEALTH	Children's Global Assessment Scale (CGAS)	4–18 years, Children and young people in clinical care	Global functioning	Proxy	1 item	Free	Krause et al. (2021)
	HEADS-ED	Children and young people representing to emergency departments	Mental health screening tool, focus on psychosocial functioning and history	Proxy	Includes seven psychosocial variables	Free	Newton et al., 2017
	Paediatric Symptom Checklist and Preschool Paediatric Symptom Checklist (PSC)	4–16 years (PSC), Children and young people in mental health services, Care experienced children and young people	Assessment measure, Psychosocial problems and overall functioning (including school and peer relationships)	Proxy (caregiver)	35 items	Free	Zima et al. (2019), McCrae and Brown (2017) and Becker-Haimes et al. (2020)
	Devereux Early Childhood Assessment (DECA-C)	Children 2–5 years	Social/emotional development	Proxy	62 items	Not free	Halle and Darling-Churchill (2016)
	Social Skills Rating System (SSRS)	Children 0–5 years	Social/emotional development	Proxy and self	34 to 57 items	Not free	Halle and Darling-Churchill (2016)
	Infant Toddler Social Emotional Assessment (ITSEA)	Children age 3 and less	Social/emotional development	Proxy	166–174 items	Not free	Halle and Darling-Churchill (2016)
	Ohio Scales	5–18 years	Overall mental health	Proxy and Self	48 items	Free	Becker-Haimes et al. (2020)
	Strength and Difficulty Questionnaire (SDQ)	3–16 years, Community mental health settings, Care experienced children and young people	Assessment measure, Prosocial behaviour and psychopathology	Proxy and Self	25 items	Free	Becker-Haimes et al. (2020) and McCrae and Brown (2017)
	Young Person’s Clinical Outcomes in Routine Evaluation (YP-CORE)	11–16 years	Outcome measure for psychological recovery	Proxy and self	10 items	Free	Becker-Haimes et al. (2020)
	The Ages and Stages Questionnaire: Social-Emotional (ASQ-SE)	3 months to 5,5 years, Care experienced children	Social-emotional development	Proxy	19–33 items		McCrae and Brown (2017)
	Brief Infant–Toddler Social and Emotional Assessment (BITSEA)	1 to 3 years, Care experienced children	Social-emotional and behaviour problems	Proxy	42 items		McCrae and Brown (2017)
	Preschool and Kindergarten Behavior Scales (PKBS-2)	3–6 years, Care experienced children	Social skills and behaviour problems	Proxy	76 items		McCrae and Brown (2017)
	Eyberg Child Behavior Inventory (ECBI)	2–16 years, Care experienced children	Conduct and disruptive behaviour	Proxy	36 items		McCrae and Brown (2017)
	Internalizing Symptoms Scale for Children (ISSC)	8–12 years, Care experienced children	Negative affect (depressive, anxiety symptoms)	Self	48 items		McCrae and Brown (2017)
QUALITY OF LIFE, HEALTH RELATED QUALITY OF LIFE AND WELLBEING	KIDSCREEN	8–18 years General population, Youth with disabilities, Validated in children with ADHD	Covers wellbeing, emotions, cognition, social relationships (home and school), autonomy and activities. For initial assessments, as well as interventions (change)	Self and proxy-report	10	Free	Krause et al. (2021), Davis et al. (2018) and Mierau et al. (2020)
	KINDL	4–17 years (self) 3–17 years (parent)	Wellbeing	Self and proxy-report	24	Free	Davis et al. (2018)
	Paediatric Quality of Life (PedsQL)	8–18 years, Poor quality in use with young children (Mierau et al., 2020), Validated with autistic youth	Covers emotional, social and school functioning	Self and proxy-report	29	Not free	Ikeda et al. (2014) and Mierau et al. (2020)
	Child Health and Illness Profile (CHIP)	6–11 years (self and proxy) 11–17 years (self)	Covers satisfaction, comfort, disorders, risks, resilience, achievement	Self and proxy-report	45, 76 or 153		Mierau et al. (2020)
	Child Health Questionnaire (CHQ)	5–18 years (parent) 10–18 (self)	Covers physical, emotional, behaviour, family domains	Self and proxy-report	28, 50 or 87	Free	Mierau et al. (2020)
ANXIETY AND DEPRESSION	Revised Children's Anxiety & Depression Scale (RCADS)	6–18 years	Anxiety and Depression	Self and proxy-report	25	Free	Krause et al. (2021), Lecavalier et al. (2014), Becker-Haimes et al. (2020) and Wigham and McConachie (2014)
	Children's Anxiety Life Interference Scale (CALIS)	6–17 years	To assess how anxiety affects functioning at home, school and other environments	Self and proxy-report	9 or 10	Free	Krause et al. (2021)
	Anxiety Disorder Interview Schedule (ADIS)	6–18 years, Used with autistic youth and youth with developmental disabilities	Anxiety	Clinician interview parent and child		Not free	Grondhuis and Aman (2012) and Lecavalier et al. (2014)
	Child and Adolescent Symptom Inventory (CASI)	5–18 years, Used with autistic youth and youth with developmental disabilities	Emotional and behavioural disorders	Proxy	125 or 173	Not free	Lecavalier et al. (2014)
	Paediatric Anxiety Rating Scale (PARS)	6–17 years, Used with autistic youth and youth with developmental disabilities	Anxiety	Clinician interview		Not free	Lecavalier et al. (2014)
	Multidimensional Anxiety Scale for Children (MASC)	8–19 years, Used with autistic youth and youth with developmental disabilities	Anxiety	Self and proxy	39		Lecavalier et al. (2014)
	Screen for Child Anxiety Related Emotional Disorders (SCARED)	6–17 years	Anxiety	Self and proxy	41	Free	Lecavalier et al. (2014), Wigham and McConachie (2014) and Becker-Haimes et al. (2020)
	Spence Children’s Anxiety Scale (SCAS)	8–15 years, Children in the general population and autistic youth	Anxiety	Self and proxy	44	Free	Becker-Haimes et al. (2020), Wigham and McConachie (2014)
	Anxiety, Depression and Mood Scale (ADAMS)	10–80 years, Used with autistic youth and youth with developmental disabilities	Anxiety and Depression	Proxy	28		Lecavalier et al. (2014)
	Beck Depression Inventories-Youth Subscale (BDI-Y)	7–18 years	Depression	Self	20	Free	Carnevale (2011)
	Center for Epidemiological Studies-Depression Scale for Children (CES-D)	6–17 years	Depression	Self	20	Free	Carnevale (2011)
	Positive and Negative Affect Scale for Children (PANAS-C)	Children	Positive and negative emotions	Self	10 or 29		Becker-Haimes et al. (2020)
	Patient Health Questionnaire-9 (PHQ-9)	Children and adults	Depression	Self	9	Free	Becker-Haimes et al. (2020)
	Mood and Feelings Questionnaire (MFQ)	6–17 years	Depression	Self and proxy	33	Free	Becker-Haimes et al. (2020)
SOCIAL ANXIETY OR PHOBIA	The social phobia and anxiety inventory for children (SPAI-C)	8–17 years	Cognitive, behaviour and somatic domains	Self and proxy	26		Tulbure et al. (2012)
	Social Anxiety Scale for Adolescents (SAS-A)	5–18 years	Cognitive, behaviour, subjective domains	Self and proxy	22		Tulbure et al. (2012)
	Social Phobia Inventory (SPIN)	13–18 years	Behaviour, somatic, subjective domains	Self	17		Tulbure et al. (2012)
	Liebowitz Social Anxiety Scale (LSAS-CA)	8–18 years	Behaviour, somatic, subjective domains	Self	24		Tulbure et al. (2012)
NEGATIVE EMOTIONS	The Child-Adolescent Perfectionism Scale (CAPS)	Children and adolescents	Perfectionism	Self	18		Ashra et al. (2021)
	Children’s automatic Thoughts Scale (CATS)	8–17 years	Negative self-cognition	Self	40	Free	Ashra et al. (2021)
MANIA	Child Mania Rating Scale-Parent (CMRS-P)	9–17 years	Mania	Proxy	21	Free	Becker-Haimes et al. (2020)
PTSD	Children's Revised Impact of Events Scale (CRIES)	7–18 years, General population	To assess change in outcomes over time, NOT for diagnosis or thorough clinical assessments	Self and proxy-report	8 or 13	Free	Krause et al. (2021)
	Children's Post-Traumatic Cognitions Inventory (CPTCI)	6–18 years	Traumatic stress	Self			Becker-Haimes et al. (2020)
OCD	Obsessive–Compulsive Inventory for Children (OCI-CV)	6–18 years, General population	To assess change in outcomes over time, NOT for diagnosis or thorough clinical assessments	Self-report	21	Free	Krause et al. (2021)
	Children’s Yale-Brown Obsessive Compulsive Scale (CY-BOCS)	5–17 years	OCD symptoms	Self and proxy	10	Free	Bennett et al. (2017)
SUICIDE/ SELF-HARM	Columbia Suicide Severity Rating Scale (C-SSRS)	12–18 years, General population	Suicide risk	Self-report	3 or 6	Free	Krause et al. (2021) and Carter et al. (2019)
	Ask Suicide Screening Questions (ASQ)	Children and adults	Suicide risk	Self	4	Free	Newton et al. (2017)
	Alexian Brothers Urge to Self-Injure Scale (ABUSI)	Children and adults	Cognitive and emotional domains to assess risk of self-injurious behaviour	Self	5	Free	Becker-Haimes et al. (2020)
SUBSTANCE USE	CRAFFT	12–21 years	Substance use and related problems	Self	6	Free	Pilowsky and Wu (2013)
	DSM-IV (two-items)				2		Newton et al. (2017)
DISRUPTIVE BEHAVIOUR	ASEBA	2–18 years	Problem behaviour	Self and proxy	113	Not free	Erford et al. (2018)
	Conners-3	6–18 years	Assessment of ADHD, oppositional defiant disorder and conduct disorder	Self and proxy	39 or 43	Not free	Erford et al. (2018)
	IOWA Conners	Children	Inattention, Impulsivity, Oppositional-defiant behaviours	Proxy	10	Free	Becker-Haimes et al. (2020)
	Child Behaviour Checklist (CBCL)	1,5 – 5 years (99 items) 6–18 years (118 items)	Behaviour and emotion problems	Self and proxy	99 or 118	Free	Hanratty et al. (2015)
	The Home Situations Questionnaire—Pervasive Developmental Disorders (HSQ-PDD)	3–14 years, autistic children and children with developmental disability	Behaviour non-compliance in everyday life	Proxy	25		Hanratty et al. (2015)
	Vanderbilt ADHD Teacher Rating Scale (VADTRS)		ADHD symptoms, anxiety, depression, oppositional-defiant behaviours	Proxy			Becker-Haimes et al. (2020)
	SNAP-IV	6–18 years	ADHD and ODD symptoms	Proxy	90		Becker-Haimes et al. (2020)
	Strengths and Weaknesses of ADHD-symptoms and Normal-behaviors (SWAN)		ADHD symptoms (including positive ones)	Proxy	30		Becker-Haimes et al. (2020)
**disordered eating**	Bulimic Investigatory Test, Edinburgh (BITE)		Symptoms and severity of binge-eating	Self			Becker-Haimes et al. (2020)
	Eating Disorder Diagnostic Scale (EDDS)	13–65 years	Anorexia, bulimia, binge-eating domains	Self	22		Becker-Haimes et al. (2020)
